# The impact of maternal RSV vaccine to protect infants in Gavi-supported countries: Estimates from two models

**DOI:** 10.1016/j.vaccine.2020.06.036

**Published:** 2020-07-14

**Authors:** Ranju Baral, Xiao Li, Lander Willem, Marina Antillon, Alba Vilajeliu, Mark Jit, Philippe Beutels, Clint Pecenka

**Affiliations:** a PATH, PO Box 900922, Seattle, WA, 98109, USA; b Centre for Health Economics Research & Modelling Infectious Diseases (CHERMID), Vaccine & Infectious Disease Institute, Campus Drie Eiken, Universiteitsplein 1 – 2610, Wilrijk, Belgium; c University of Basel, Klingelbergstrasse 61, 4056 Basel, Switzerland; d Swiss Tropical and Public Health Institute, Socinstrasse 57, 4051 Basel, Switzerland; e Independent consultant, 3073 Cleveland Ave NW, Washington, DC 20008, USA; f Department of Infectious Disease Epidemiology, London School of Hygiene & Tropical Medicine, Keppel Street, London WC1E 7HT, United Kingdom; g Modelling and Economics Unit, Public Health England, 61 Colindale Avenue, London NW9 5EQ, United Kingdom; h School of Public Health, Patrick Manson Building, 7 Sassoon Road, The University of Hong Kong, Hong Kong Special Administrative Region

**Keywords:** Respiratory syncytial virus (RSV), Maternal RSV vaccine, Child health, Gavi, the Vaccine Alliance, Health impact

## Abstract

**Background:**

Interventions to protect young infants against respiratory syncytial virus (RSV) are in advanced phases of development and are expected to be available in the foreseeable future. Gavi, the Vaccine Alliance, included maternal vaccines and infant monoclonal antibodies for RSV as part of the 2018 vaccine investment strategy (VIS) and decided to support these products subject to licensure, World Health Organization prequalification, Strategic Advisory Group of Experts recommendation, and meeting the financial assumptions used as the basis of the investment case. Impact estimates reported in this manuscript were used to inform the Gavi VIS.

**Methods:**

We compared two independent vaccine impact models to evaluate a potential maternal RSV vaccine’s impact on infant health in 73 Gavi-supported countries. Key inputs were harmonized across both models. We analyzed various scenarios to evaluate the effect of uncertain model parameters such as vaccine efficacy, duration of infant protection, and infant disease burden. Estimates of averted cases, severe cases, hospitalizations, deaths, and disability-adjusted life years (DALYs) were calculated over the 2023–2035 horizon.

**Findings:**

A maternal RSV vaccine with 60% efficacy offering 5 months of infant protection implemented across 73 low- and middle-income countries could avert 10.1–12.5 million cases, 2.8–4.0 million hospitalizations, 123.7–177.7 thousand deaths, and 8.5–11.9 million DALYs among infants under 6 months of age for the duration of analysis (2023–2035). Maternal RSV vaccination was projected to avert up to 42% of estimated RSV deaths among infants under 6 months in year 2035. Alternative scenario analyses with higher disease burden assumptions showed that a maternal vaccine could avert as many as 325–355 thousand deaths among infants under 6 months.

**Interpretation:**

RSV maternal immunization is projected to substantially reduce mortality and morbidity among young infants if introduced across Gavi-supported countries.

**Funding:**

This work was supported by Bill & Melinda Gates Foundation, Seattle, WA, and Respiratory Syncytial Virus Consortium in Europe. The findings and conclusions contained within are those of the authors and do not necessarily reflect positions or policies of the Bill & Melinda Gates Foundation or of the Respiratory Syncytial Virus Consortium. LW is supported by Research Foundation–Flanders (1234620 N).

## Highlights

• First study examining potential impact of a maternal RSV vaccine across LMICs.
• Results from independent models to inform Gavi’s 2018 Vaccine Investment Strategy.
• Experts and stakeholders advised on methods, assumptions, and key model inputs.
• Substantial potential to reduce infant morbidity and mortality in Gavi countries.

## 1 Introduction

Under-5 mortality declined by 59% globally between 1990 and 2018 [1]. Despite this impressive reduction, the pace of decline has not been as rapid for infants and neonates. In 2018, approximately 70% and 47% of deaths in children under 5 years of age occurred among infants and neonates, respectively [1]. Addressing disease burden among this age group is imperative to achieving the Sustainable Development Goal of ending preventable deaths in newborns and children under 5 years of age [1], [2].

A significant proportion of morbidity and mortality during the first year of life is due to infectious diseases [3], [4]. Although many of these diseases are vaccine preventable, protecting a newborn with active immunization is not always possible as the newborn’s immune response system continues to develop and particularly when multiple doses are required over weeks or months [5]. One alternative to combat neonatal and early infant mortality is to vaccinate pregnant women. Most adults have been primed by natural infection with respiratory syncytial virus (RSV). Upon vaccination the mother’s antibodies are boosted and are transferred to the fetus to provide protection during the infant’s first months of life. Maternal immunization (MI) is gaining support, especially following the success of MI strategies to reduce the burden of neonatal tetanus, infant pertussis, and maternal and infant influenza [6].

RSV, a common cause of acute lower respiratory infections (ALRIs), disproportionately impacts infants in their first months of life [3], [4]. Globally, RSV-associated ALRIs are responsible for an estimated 118,000 annual deaths among children under 5 years [7]. About 46% of those deaths are estimated to occur among children<6 months of age [7]. This burden predominantly affects children in low- and middle-income countries (LMICs), where approximately 90% of RSV-associated ALRI mortality occurs. Current preventive and treatment options for RSV are costly, but multiple RSV interventions are in development with licensure of maternal vaccines and long-lasting infant monoclonal antibodies (mAbs) anticipated in the coming years [8].

Some of the RSV interventions under development, including vaccines for pregnant women and infant mAbs, were considered by Gavi, the Vaccine Alliance, in its 2018 Vaccine Investment Strategy (VIS) for the 2021–2025 funding period. The VIS occurs every five years and allows Gavi to prioritize new investments for its next strategic and funding cycle. Health impact as measured by cases and deaths averted is one of the main criteria Gavi uses to prioritize interventions. This paper presents model-based estimates of the potential health impact of RSV maternal immunization (RSV MI) in Gavi-supported countries, which were generated independently to inform Gavi’s decision-making on maternal RSV vaccination in its funding portfolio. Gavi’s VIS analysis for RSV interventions included multiple phases of evaluation of assumptions, scenarios, and independent modeling outputs by expert groups from June 2017 to June 2019. The scenarios and results discussed in this paper include those prepared for the VIS as well as those considering alternative disease burden assumptions based on expert input regarding emerging yet unpublished data and recent vaccine clinical trial results that were not available in time to inform the VIS. Importantly, the results presented here only represent impact on RSV in young infants and do not incorporate the substantial impact vaccination would likely have on all infant lower respiratory tract infections, as suggested by recent studies [9], [10].

## 2 Methods

Several models of RSV MI were independently developed by distinct groups to allow for cross-validation and to assess the potential impact of uncertainty related to model structure choices. To guide model development, the Gavi Secretariat convened a series of consultations and discussion meetings with select expert groups and stakeholders (see Appendix Table 1 for full list) to assess and align on methods, assumptions, data inputs, country vaccine introduction scenarios, and sensitivity analyses. Each modeling group then separately analyzed the impact of RSV MI based on the common set of inputs in order to inform Gavi’s assessment of RSV as part of the VIS. This paper includes results from two of the RSV MI models (PATH and the University of Antwerp in partnership with the London School of Hygiene & Tropical Medicine [hereafter referred to as UA]) and a subset of scenarios used to inform the Gavi 2018 VIS.

### 2.1 Model

Both RSV MI models are static cohort models that follow a common structure (Appendix Fig. 1) and compare the annual impact of vaccinating pregnant women against RSV to protect infants up to 6 months of age compared to an alternative of no RSV MI. We assumed single-dose year-round vaccination of pregnant women during established antenatal care (ANC) visits. All pregnant women attending ANC between 24 and 36 weeks of gestation were assumed eligible for vaccination.

Common model outcomes were non-severe episodes, hospitalizations, deaths, and disability-adjusted life years (DALYs) averted among infants by vaccinating pregnant women across 73 LMICs between 2023 and 2035. The PATH model also estimated severe episodes of RSV among infants. The year of introduction for each country was based on product development, global policy timeline projections at the time of the analysis, and Gavi country introduction year assumptions for vaccines under consideration in the VIS. In order to compare results across potential additions to Gavi’s investment portfolio, the health outcome estimates presented in this paper are not discounted.

### 2.2 Key data inputs and assumptions

The modeling groups harmonized key model parameters to support comparability of results, including demography, vaccine characteristics, estimated country introduction dates, and vaccine delivery scenarios. A list of shared input parameters and sources is provided in Table 1. Modeling groups used the same source for disease burden data but made different methodological decisions about how to incorporate these data to account for insufficient granularity and source data presentation that diverged from model input requirements.

**Table 1:** Primary inputs harmonized across models.

Input	Value	Sources
Target population by country	Approximately 90 million pregnant women globally, per year	United Nations Population Database, 2017 and Lawn et al., 2016 [11], [12]
Vaccine schedule	Single dose provided year-round as part of ANC services	Preferred Product Characteristics [13]; expert opinion
Vaccination window	24–36 weeks of gestation	Preferred Product Characteristics [13]; expert opinion
Vaccine coverage by country	Average in year 2023, 69% (21%–96%)	DHS and WHO [14], [15]
Vaccine introduction dates by country	Phased, 2023 to 2035	Product development timeline [8]; prior vaccine introduction
Vaccine efficacy for infant protection	Midpoint 60%	Preferred Product Characteristics [13]; expert opinion
Duration of protection	Midpoint 5 months, no waning before 5 months	Preferred Product Characteristics [13]; expert opinion

Abbreviations: ANC, antenatal care; DHS, Demographic and Health Surveys; WHO, World Health Organization.

#### 2.2.1 Disease burden

Both models estimated RSV infant disease burden based on a recent systematic review [7]. Key burden inputs included rates of incidence, severe incidence, hospitalizations, and mortality, as defined in the literature. Country-specific estimates of RSV incidence among children 0 to 5 years were used for 63 of 73 countries (86%) included in the analysis. Where country-specific data were not available, the PATH model applied an average of the estimates from other countries in the same income group per World Bank’s definition and within the same geographic region [16]. The UA model bridged the estimates from countries in the same geographic regions with similar infant and under-5 mortality rates (Appendix Table 3.3).

While both models relied on the same data source for input parameters, there were key differences in the application of these parameters (Appendix Table 2). For example, age-stratified incidence estimates in the literature have insufficient age granularity to discern meaningful differences by age group as well as by region/country. Each modeling group addressed this challenge separately—the UA model fitted a spline to interpolate the age-specific data, whereas the PATH model assumed constant incidence within age classes. Both models applied country-specific rates to estimate cases among children 0 through 5 years (see Appendix Table 2).

Disease burden estimates, especially in low-income settings, are generally derived from hospital-based data [7]. However, the number of unreported RSV cases and deaths in the community is largely unknown [17]. Both models therefore adjusted for unreported community deaths using mortality multipliers as suggested in the literature [7]. Input parameter values for disease burden utilized by each model are presented in Table 2.

**Table 2:** Input parameters for RSV disease burden in children.

Parameter	UA	PATH	Source
Annual incidence of RSV-ALRI per 1,000 children under 5	Country-specific estimates (35.3 to 65.6)	Country-specific estimates (35.3 to 65.6)	[7, Supplementary Table 18*]
Annual incidence of RSV-ALRI per 1,000 children under 5, by age group	Developing country estimate	Developing country estimate	[7, supplementary Table 9*]
0–27 days	40.0	40.0	
28–< 3 months	45.7	45.7	
3–5 months	99.6	99.6	
6–11 months	98.8	98.8	
12–23 months	79.1	79.1	
Annual incidence of severe RSV-ALRI per 1,000 children under 5, by age group	Not included due to insufficient age-specific data	Developing country estimate	[7, Table 1*]
0–5 months	NA	36.1	
6–11 months	NA	24.7	
0–59 months	NA	10.2	
Annual hospital admissions for RSV-associated ALRI per 1,000 children under 5, by age group	Developing country estimate	Developing country estimate	[7, Supplementary Table 20 for UA estimate, and Table 1 for PATH estimate*]
0–5 months	0–27 days: 15.9; 28 days–3 months: 26.1; 3–5 months: 20.7	20.2	
6–11 months	6–8 months: 12; 9–11 months: 11.3	11	
Hospital case fatality risk (%), by age group	Developing country estimate	Developing country estimate	[7, Supplementary Table 20 for UA estimate, and Table 2 for PATH estimate*]
0–5 months	0–27 days: 5.3; 28 days–3 months: 2.3; 3–5 months: 2.4	2.2	
6–11 months	6–8 months: 3.0; 9–11 months: 3.6	2.4	
Disability weight for DALY calculation			[18]
Severe ALRI	0.21	0.21	
Non-severe ALRI	0.053	0.053	
Duration of illness (days)	11.2	11.2	[19]

Abbreviations: ALRI, acute lower respiratory infection; DALY, disability-adjusted life year; NA, not applicable; RSV, respiratory syncytial virus; UA, University of Antwerp in partnership with the London School of Hygiene & Tropical Medicine. Table numbers represent tables from the source literature [7] from where the input values were derived.

#### 2.2.2 Vaccine characteristics

Vaccine characteristics including the duration of protection in infants and vaccine efficacy used for modeling were informed by WHO’s RSV maternal vaccine Preferred Product Characteristics (PPC) with additional perspectives from a group of RSV experts coordinated by Gavi to inform the vaccine investment strategy. WHO PPC indicate a vaccine with 50% efficacy and duration of protection of 3 months as acceptable and a product with >70% efficacy and > 4 months as preferred. For the baseline scenario, we used a 5 months duration of protection and 60% vaccine efficacy and evaluated a range of values to test the impact of uncertain input parameters in the scenario analysis.

#### 2.2.3 Target population, coverage, and country vaccine introduction

Both models used the same target population and coverage rate for each country. The number of pregnant women eligible for vaccination, by country, was derived from the World Population Prospects’ annual birth projections and country-specific projected population growth rates [12]. Annual birth projections were adjusted by country-specific stillbirth rates [11], yielding the total number of pregnant women in each country.

The RSV maternal vaccine was modeled to be delivered year-round in a single dose between 24 and 36 weeks of gestation, requiring data on the timing of ANC visits or contacts by gestational age. These data are not widely available in LMICs, so vaccine coverage estimates were modeled for each country based on reported ANC coverage, the projected timing of ANC visits, and the probability of receiving ANC services. Coverage levels were estimated in 2017 and projected to increase in subsequent years to be consistent with modeling for childhood vaccines being considered for Gavi’s portfolio. We assumed that coverage increased by three percentage points per year up to 70%, after which it increased by one percentage point per year until reaching 95%, consistent with other vaccine models considered in the VIS [20]. The Appendix Fig. 2 details steps used to model eligibility and vaccination coverage during the vaccination window.

Both models assumed the vaccine would be available, with no supply constraint, for introduction in Gavi-supported countries by 2023 based on the status of clinical studies of lead vaccine candidates at the time of analysis and accounting for time required for licensure and World Health Organization (WHO) prequalification [8]. Introduction of the vaccine across 73 Gavi-supported/formerly supported countries (hereafter “Gavi countries”) was modeled to be phased between 2023 and 2035. A description of introduction timelines is available in the Appendix Table 3.2.

#### 2.2.4 Sensitivity and scenario analysis

There is wide uncertainty around various input parameters, particularly disease burden and vaccine characteristics like efficacy and duration of protection. To address this challenge, we conducted a series of one-way sensitivity analyses where we varied selected model parameters across a plausible range to assess each parameter’s influence on model results. Both models used common uncertainty ranges for each parameter to conduct model sensitivity. Maternal vaccine efficacy was modeled to a range between 30% and 90% and duration of infant protection was modeled to last between 4 and 6 months. We also conducted scenario analyses where vaccine efficacy and duration of protection were simultaneously varied to evaluate the potential combined impact on health outcomes. Additionally, we estimated the impact of MI under a high disease burden scenario as suggested by recent surveillance studies [21], [22]. Finally, as an adjunct to the information provided to Gavi, we formed an additional scenario using vaccine efficacy data from the Phase 3 trial of a lead maternal vaccine candidate [9]. A list of all scenarios and their descriptions is provided in Table 3. A subset of these scenarios was used to inform the Gavi VIS. In particular, Gavi assumed a vaccine efficacy range of 50%–90% and corresponding duration of protection of 3 to 6 months. Additionally, Gavi prioritized model results for the subset of countries that were projected to remain eligible for new vaccine support when RSV interventions are available. Also, Gavi used a pooled analysis that included the third model not included in this manuscript [23].

**Table 3:** List of scenarios modeled.

#	Scenario	Vaccine efficacy	DOP (months)	Disease burden	Source
1	Baseline (VE 60%, DOP 5 m)	60% (constant for all outcomes)	5	[7]	Assumptions
2	Baseline VE (60%) and minimum DOP (4 m)	60% (constant for all outcomes)	4	[7]	Assumptions
3	Baseline VE (60%) and maximum DOP (6 m)	60% (constant for all outcomes)	6	[7]	Assumptions
4	Minimum (VE 30%, DOP 4 m)	30% (constant for all outcomes)	4	[7]	Assumptions
5	Minimum VE (30%) and baseline DOP (5 m)	30% (constant for all outcomes)	5	[7]	Assumptions
6	Minimum VE (30%) and maximum DOP (6 m)	30% (constant for all outcomes)	6	[7]	Assumptions
7	Maximum (VE 90%, DOP 6 m)	90% (constant for all outcomes)	6	[7]	Assumptions
8	Maximum VE (90%) and minimum DOP (4 m)	90% (constant for all outcomes)	4	[7]	Assumptions
9	Maximum VE (90%) and baseline DOP (5 m)	90% (constant for all outcomes)	5	[7]	Assumptions
10	Prepare trial primary results VE and DOP	Cases = 39.4%; Hospitalization = 44.4%; Death = 48.3%	3	[7]	Prepare trial results (Phase 3), based on *primary* analysis that uses per protocol population [9]
11	Prepare trial expanded results VE and DOP	Cases = 40.9%; Hospitalization = 41.7%; Death = 59.6%	3	[7]	Prepare trial results (Phase 3), based on *expanded* analysis that includes additional population excluded per protocol [9]
12	Prepare trial primary results VE and DOP, under high burden	Cases = 39.4%; Hospitalization = 44.4%; Death = 48.3%	3	8.7% of all LRTI deaths attributed to RSV, as suggested by new evidence [21], [22]	Prepare trial results (Phase 3), based on *primary* analysis that uses per protocol population [9]
13	Prepare trial expanded results VE and DOP, under high burden	Cases = 40.9%; Hospitalization = 41.7%; Death = 59.6%	3	8.7% of all LRTI deaths attributed to RSV, as suggested by new evidence [21], [22]	Prepare trial results (Phase 3), based on *expanded* analysis that includes additional population excluded per protocol [9]
14	Baseline VE and DOP, under high burden	60% (constant for all outcomes)	5	8.7% of all LRTI deaths attributed to RSV, as suggested by new evidence [21], [22]	Base case vaccine efficacy and duration of protection under a high burden scenario

Abbreviations: DOP, duration of protection; VE, vaccine efficacy; LRTI, lower respiratory tract infection; m, month.

## 3 Results

Projected health outcomes averted among infants under 6 months of age after the introduction of RSV MI in 73 Gavi countries between 2023 and 2035 are provided in Table 4, by model and averaged across both models. In the baseline scenario, the maternal RSV vaccine is estimated to avert an average of 11.3 million RSV cases (inter-model range: 10.1 to 12.6 million). Additionally, we project that more than 3.4 million hospitalizations (inter-model range: 2.8 to 4.0 million), 150 thousand deaths (inter-model range: 124 to 178 thousand), and 10.3 million DALYs (inter-model range: 8.6 to 12.0 million) would be averted across all years. By 2035, on average, we project that 41% to 42% of annual RSV deaths in infants under 6 months in the 73 countries would be averted. In a cohort of 100,000 vaccinated pregnant women, the models predict prevention of approximately 1,900 cases (inter-model range: 1,700 to 2,100) of RSV and 25 infant deaths (inter-model range: 21 to 30).

**Table 4:** Estimates of health impacts in 73 Gavi countries in year 2023–2035 for the baseline scenario.

RSV associated	UA	PATH	Average across models
Total RSV cases averted^	10,106,070 (3,493,773–20,811,437)	12,553,081 (4,385,604–24,456,864)	11,329,575
Non-severe cases averted	6,053,139 (1,880,906–13,775,109)	8,868,303 (2,911,693–18,173,349)	7,460,721
Severe cases averted	NA	3,684,778 (1,473,911–6,283,516)	3,684,778
Hospital admissions averted	4,052,931 (1,612,867–7,036,328)	2,840,087 (1,136,035–4,724,105)	3,446,509
Deaths averted	177,717 (70,418–309,811)	123,714 (49,486–207,619)	150,716
YLDs averted	35,963 (13,439–67,740)	37,369 (13,914–68,707)	36,666
YLLs averted	11,927,676 (4,724,075–20,806,417)	8,520,783 (3,408,313–14,299,729)	10,224,230
DALYs averted*	11,963,639 (4,737,529–20,874,133)	8,558,152 (3,422,227–14,368,437)	10,260,896
Number needed to vaccinate to avert a death	3,339 (1,915–8,426)	4,796 (2,858–11,990)	4,067
Infant cases averted per 100,000 vaccinated pregnant women	1,703 (589–3,507)	2,116 (739–4,122)	1,909
Infant deaths averted per 100,000 vaccinated pregnant women	30 (12–52)	21 (8–35)	25
Proportion of RSV deaths averted among children < 6 months (total)	0.27 (0.11–0.46)	0.27 (0.11–0.46)	0.27
Proportion of RSV deaths averted among children < 6 months (in 2035)	0.41 (0.16–0.71)	0.42 (0.17–0.70)	0.42

Abbreviations: DALY, disability-adjusted life year; NA, not applicable; RSV, respiratory syncytial virus; UA, University of Antwerp in partnership with the London School of Hygiene & Tropical Medicine; YLD, years lived with disability; YLL, years of life lost.

Baseline scenario applied vaccine efficacy of 60% and 5 months duration of protection. Values in parenthesis represent estimates from minimum (Scenario 4) and maximum (Scenario 7) scenarios.

^ UA model applied: Total RSV cases = non-severe cases + hospital admissions. PATH model applied: Total RSV cases = non-severe + severe cases.

* Undiscounted DALYs are used.

**Fig. 1:**
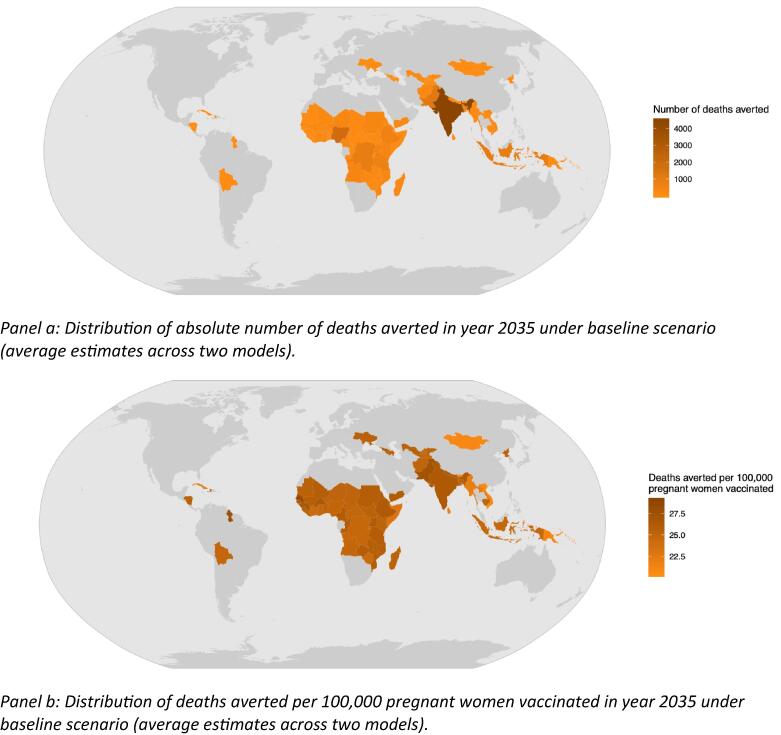
Distribution of health impact of RSV MI in year 2035, under baseline scenario. Panel a: Distribution of absolute number of deaths averted in year 2035 under baseline scenario (average estimates across two models). Panel b: Distribution of deaths averted per 100,000 pregnant women vaccinated in year 2035 under baseline scenario (average estimates across two models).

Distribution of total deaths averted, and the number of deaths averted per 100 thousand vaccinated pregnant women, under the baseline scenario, for year 2035, is shown in Fig. 1. Although countries with larger birth cohorts (for example, India) are predicted to have a larger impact in terms of absolute number of deaths averted (panel a), impact of vaccination that takes into account the relative coverage (defined as deaths averted per 100 thousand vaccinated women) reflects a more even distribution of impact (panel b). Appendix Tables 4.1–4.3 contain country-specific estimates of disease burden and expected health impact of the vaccine.

**Fig. 2:**
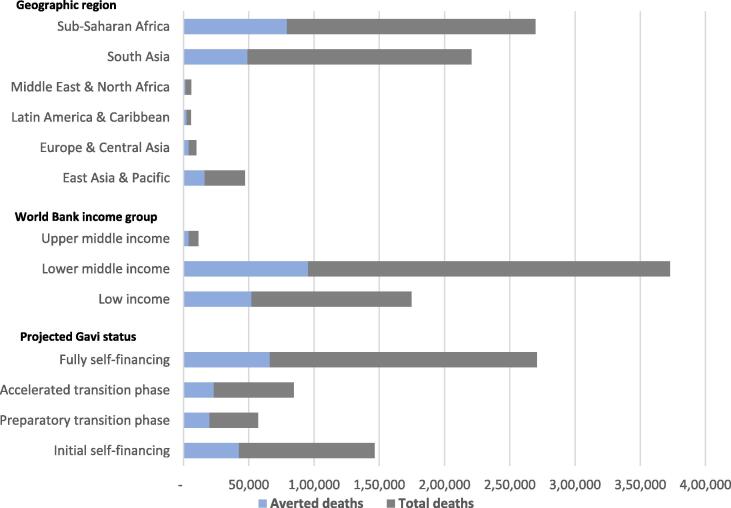
Average number of estimated deaths and deaths averted among < 6 months old (2023–2035) by region, income group, and Gavi eligibility status.

Fig. 2 shows the average predicted number of deaths due to RSV among children under 6 months and the estimated average deaths averted due to RSV MI stratified by region, World Bank income group, and country’s expected Gavi transition status [24]. We found that more than 80% of the vaccine impact would occur among countries in sub-Saharan Africa and South Asia, the regions that comprise the largest estimated disease burden as well as countries receiving Gavi support. When average model results were stratified based on each country’s projected Gavi financing status in 2023, we find more than a quarter (28%) of the impact is expected among countries in the initial self-financing phase, 13% in the preparatory transition phase, and 15% in the accelerated transition phase. Since many of the countries included in our analysis are expected to transition from Gavi support in the later years of the analysis, much of the impact (43%) would be among fully self-financing countries that no longer receive Gavi support.

### 3.1 Sensitivity and scenario analysis

We conducted one-way sensitivity analyses to assess the impact of individual parameters on our estimates of health impact. Given that vaccine efficacy and duration of infant protection are uncertain, we modeled a range of scenarios to understand the potential impact on model outcomes. Additional scenarios using latest clinical trial data [9] and higher disease burden were also modeled (see Table 3 for scenario description).

**Fig. 3:**
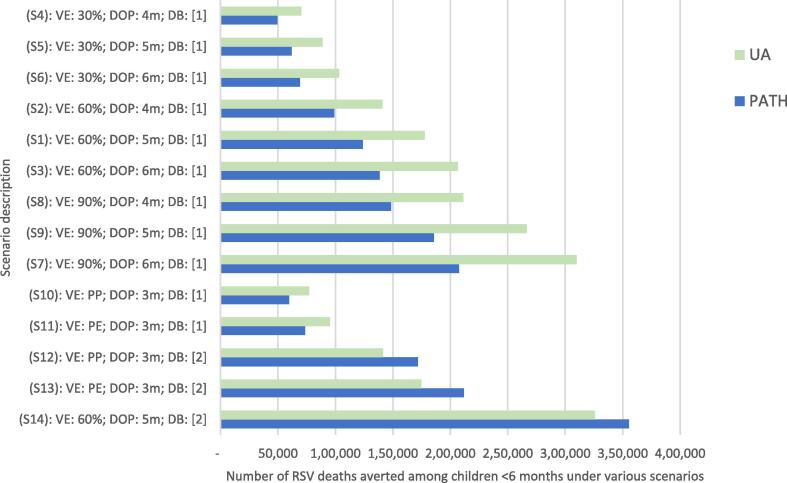
One-way sensitivity analysis. Abbreviations: DB, disease burden (source 1 = Shi et al., 2017 [7]; source 2 = 8.7% of all LRTI deaths attributed to RSV, as suggested by new evidence [9]); DOP, duration of protection; m, month; PE, Prepare trial expanded efficacy results; PP, Prepare trial primary endpoint efficacy; RSV, respiratory syncytial virus, S, scenario; VE, vaccine efficacy.

Fig. 3 shows the averted disease burden among infants achieved by vaccinating pregnant women over different assumptions of efficacy and duration of protection and scenarios. The estimated burden averted monotonically increases with higher efficacy and longer duration of protection. For example, assuming 90% efficacy with 6 months of protection (Scenario 7) is estimated to result in more than four times higher impact in terms of deaths averted compared to a vaccine with 30% efficacy and 4 months of protection (Scenario 4), and roughly twice the impact compared to 60% efficacy and 5 months of protection (Scenario 1, baseline). Compared to the baseline scenario (Scenario 1, baseline) deaths averted are more than double in higher mortality burden scenario (Scenario 14), ceteris paribus.

Using the Prepare™ Phase 3 clinical trial vaccine efficacy data (Scenarios 10 and 11), our models predict roughly 3.0 to 3.46 million cases averted, 1.2 to 1.6 million averted hospitalizations, and about 60,000 to 95,000 deaths averted across all countries and years considered. A maternal vaccine with efficacy values comparable to Prepare Phase 3 trial results is projected to achieve about 28% to 31% of averted cases, 40% to 44% of averted hospitalizations, and 43% to 60% of averted deaths compared to the impact estimated under the baseline scenario. Detailed results under all alternative scenarios are given in Appendix Tables 4.1–4.3 and Appendix Figure 3.

## 4 Discussion

Across two independent models, we projected that introduction of RSV MI could substantially reduce the RSV burden among infants under 6 months of age across 73 Gavi countries. In our midpoint (baseline) scenario, we estimated 10 to 12 million RSV cases, 8.5 to 112 million DALYs, and 123,000 to 177,000 deaths could be averted with the maternal RSV vaccine between 2023 and 2035 across 73 Gavi countries. Results from another RSV MI impact model [23], which is consistent with the results from the models included in this study, were also used to inform the VIS. The VIS sets priorities for vaccine support programs in Gavi countries in a transparent manner through evidence-based analysis and extensive consultations [20].

A key strength of this work is independent estimation by multiple modeling groups, as recommended by WHO guidelines for vaccine impact models [25], [26], [27], that allowed for the assessment of “convergent validity” of multiple model estimates. While key model inputs of vaccine characteristics and population demographics were harmonized, each modeling group independently constructed and calibrated its model. A considerable range of potential RSV MI impact estimates were generated under various scenarios. The variations between the two model outcomes were primarily due to differences in interpretation and interpolation of data on age-specific RSV-associated disease burden [7]. A multiple model comparison allowed for assessment of model consistency, which is needed to improve credibility and to inform decision-makers in a multicountry and multi-scenario analysis.

RSV MI in Gavi countries stands to prevent approximately 42% of the RSV deaths among infants under 6 months of age that would occur without the vaccine. A few studies on the impact of RSV MI indicate about 32% reduction in RSV infections and about 50% reductions in RSV hospitalizations among infants in Kenya [28], [29]. Despite the differences in underlying model and the assumptions, our estimates are similar to the estimates of RSV MI impact in LMIC settings. Our results are also comparable with the projected impact of rotavirus vaccines, for instance, which are predicted to avert 36% of rotavirus-related diarrheal deaths among under 5 children across Gavi countries [30]. While the scale of RSV impact is smaller than that for other blockbuster vaccines, this is expected given the relative burden [31]. Lower projected impact can also be viewed as a sign of progress given the success of the global health community in reducing the burden of major causes of child mortality [32]. Declines in overall child mortality and a concentration of burden among the youngest infants also points to the need to develop interventions to protect infants during the first weeks of life before active infant vaccination can provide protection.

Maternal immunization against pertussis and influenza to protect mothers and young infants has proven effectiveness to protect many newborns against these diseases in certain geographies and is a useful reference for lessons learned [33], [34], [35], [36]. In addition to demonstrated impact of maternal immunizations, other maternal vaccines are highlighting lessons that can be gleaned for preparing health systems for new maternal vaccines [36]. RSV MI might be one of the first vaccines given to pregnant women in LMICs for the primary purpose of protecting young infants. This will be an important milestone that illustrates an increasing focus on a life-course approach to vaccination. RSV MI also represents opportunities to enhance collaboration between immunization and maternal health programs amid efforts to achieve universal health coverage and the Sustainable Development Goals [36].

A lead maternal RSV vaccine candidate did not meet the study’s primary endpoint, creating uncertainty about its licensure [9]. However, using topline efficacy results from the trial [9], our analysis shows the vaccine product would likely have a large impact on RSV deaths. This suggests that modeling and clinical trial results can better inform the potential for public health impact and decision-making than clinical trial results alone.

Our analyses have a few limitations, including model structure, source data, and the scope of benefits included in our models. Both models were static cohort-based models and, thus, do not capture indirect effects such as preventing RSV infection between mothers and other household members. We assume constant RSV burden over the period of analysis.

RSV maternal vaccine coverage that needs administration during a specific gestation window is largely unknown, especially across LMICs. We therefore modeled vaccine coverage using country-specific data on ANC visit timing [14] and WHO focused antenatal care (FANC) guidance on timing of ANC visits [37]. The newer WHO guidance on ANC [15] suggests a higher number of visits/contacts than considered in the FANC model, which suggests our vaccine coverage estimates may be underestimates as the number of ANC contacts increase. Further, the impact estimates reported in this analysis do not include the full potential impact as countries are assumed to introduce vaccines in different years and earlier introduction would imply higher impact than reported here. On the other hand, our impact estimates are sensitive to annual increase in coverage assumption, especially for countries with low predicted coverage in the first year of introduction. For example, vaccine coverage for Burundi in 2023 is predicted to be about 44%, but by the end of 2035 it is predicted to reach 74%. The impact of this linear increase in vaccine coverage assumption means the share of burden averted in Burundi reaches from 15% in 2023 to about 31% in 2035. More importantly, the investments that would be required to strengthen the health systems that are able to achieve this level of coverage and therefore the stated level of vaccine impact is likely not insignificant.

Our baseline scenario assumes vaccines’ performance that exceeds that from some existing maternal vaccines against other pathogens [38] and RSV MI clinical trial data available to date [39]. However, we anticipate that a product that could be supported by donors for broader use in the LMICs will need to offer efficacy and duration of protection in infants comparable to that indicated in the PPC [13]. The estimates across multiple scenarios capture the uncertainty of unknown product characteristics. On the other hand, children with higher risk of severe RSV disease, such as prematurely born children, may benefit differently from RSV maternal vaccine compared to the full-term infants. We do not account for any potential differences in this population due to data limitations. As a result, our models potentially bias the impact in the preterm population, especially in low-income countries with higher rates of premature births [40].

There is a dearth of RSV burden data from LMICs, and though we believe we have used the best published estimates, they are subject to uncertainty. Community-based surveillance studies suggest the real burden of RSV is much higher than reported in these predominantly hospital-based studies, with RSV being associated with as much as 8% to 11% of all lower respiratory tract deaths in some countries [21], [41], [42]. In a scenario where RSV is associated with 8.7% of all causes of lower respiratory infection deaths (Scenario 14), our models estimated roughly two-to-three times the mortality averted using the primary source of disease burden data. Moreover, there is some evidence in the literature that suggests acquiring RSV disease is associated with wheezing, asthma, or other reduced pulmonary function later in life [43], [44], [45]. This link is not yet viewed as conclusive, so long-term sequelae were excluded from our analyses, as were potential maternal benefits of vaccination such as enhanced infant protection through breastfeeding, preventing inappropriate antibiotic use and thus antimicrobial resistance, reducing treatment demands on the health system, and other socioeconomic benefits of RSV prevention. Finally, recent trial data for a lead maternal RSV vaccine candidate were not fully incorporated into this analysis for Gavi VIS decision-making. While the topline trial data demonstrate a higher efficacy against severe diseases outcomes compared to our baseline analysis, they also indicate relatively lower efficacy estimates for other disease endpoints [9]. However, the current trial results available for maternal vaccine also claim significant efficacy in reducing all respiratory hospitalizations and reduction in all cause severe hypoxemia through the first 6 months of life, critical non-RSV outcomes that were not included in our model.

The modeling work presented here represents collaborative efforts to fill important knowledge gaps for global health agencies such as Gavi and WHO and country-level stakeholders that are beginning to prioritize and plan for future vaccine introductions while vaccines are still in the development pipeline. Our study demonstrates that introduction of maternal RSV MI in low- and middle-income countries would greatly reduce mortality among the youngest infants. Most importantly, this analysis was a critical input for Gavi’s decision to support RSV maternal immunization products subject to licensure, WHO prequalification, Strategic Advisory Group of Experts recommendation, and meeting the financial assumptions used as the basis of the investment case. Future research should explore the relative effectiveness as well as cost-effectiveness of the RSV MI strategy compared to the other preventive strategies against infant RSV.

## Funding

This work was supported by Bill & Melinda Gates Foundation, Seattle, WA, and Respiratory Syncytial Virus Consortium in Europe. The findings and conclusions contained within are those of the authors and do not necessarily reflect positions or policies of the Bill & Melinda Gates Foundation or of the Respiratory Syncytial Virus Consortium. LW is supported by Research Foundation–Flanders (1234620 N).

## Declaration of Competing Interest

The authors declare that they have no known competing financial interests or personal relationships that could have appeared to influence the work reported in this paper. AV is a former Senior Programme Officer at GAVI.
