# Supplementary material for: The impact of maternal RSV vaccine to protect infants in Gavi-supported countries: Estimates from two models
Source: Vaccine. 2020 Jul 14;38(33):5139–47. doi: 10.1016/j.vaccine.2020.06.036 (PMC7342012; doi:10.1016/j.vaccine.2020.06.036)
Supplement: Supplementary data 1 [file mmc1.docx]

**The impact of maternal RSV vaccine to protect infants in Gavi-supported countries: estimates from two models**

**Supplementary materials**

**Appendix Table 1. List of RSV expert group members and stakeholders consulted by Gavi for the VIS 2018.**

| **Name** | **Affiliation** |
| --- | --- |
| Daniel Feikin | World Health Organization |
| Tracey Goodman | World Health Organization |
| Emily Wootten | World Health Organization |
| Philipp Lambach | World Health Organization |
| Erin Sparrow | World Health Organization |
| Niteen Wairagkar | Gates Foundation |
| Tasleem Kachra | Gates Foundation |
| Amanda Beal | Gates Foundation |
| Clint Pecenka | PATH |
| Deborah Higgins | PATH |
| Jessica A. Fleming | PATH |
| Ranju Baral | PATH |
| Evan Simpson | PATH |
| Kate O’Brien | Johns Hopkins University (now World Health Organization) |
| Ruth Karron | Johns Hopkins University |
| Linda Eckert | University of Washington |
| Harish Nair | University of Edinburgh |
| Mark Jit | London School of Hygiene & Tropical Medicine |
| Susan McKinney | US Agency for International Development |
| Rebecca Levine | US Agency for International Development |
| Bernard Gonik | US Agency for International Development |

**Appendix Table 2. Methodological comparison in RSV disease burden estimation across models.**

| **Inputs** | **UA** | | **PATH** | | |
| --- | --- | --- | --- | --- | --- |
| General approach to age distribution of disease | Fitted spline to interpolate large interval age-specific estimates to generate monthly age estimates. | | Assumed constant incidence within each age category. | | |
| Incidence of RSV-associated ALRI | Estimated case distribution by month of age by fitting curves to data from developing country estimate. Used fitted estimates together with country-specific incidence for 0–5 years to create age-specific estimates for each country. | | Generated incidence for 0–5 years using country-specific burden estimates and applied developing country incidence case distribution by age. Age-specific incidence cases were rescaled to match country-specific incidence envelope. | | |
| Incidence of severe RSV-associated ALRI | Not included due to insufficient age-specific estimates. | | Used developing country estimates and assumed constant incidence within each age category. | | |
| Incidence of RSV-related hospitalization | Calculated the percentage of all RSV cases that were hospitalized in a generic developing country at 1-month age intervals. Percentage was applied to the age-specific incidence in each country to derive the number of hospitalized cases at 1-month age intervals. | | Used developing country estimates and assumed uniform age distribution across month within given age bands. | | |
| Hospital case fatality | Spline to interpolate to age-specific data with 1-month age resolution. | | Applied hospital case fatality risks for developing countries to the hospitalized cases estimated above. | | |
| Adjustment for projected decline in all -cause mortality | Country-specific projected mortality rate during the study period^§^ | | Assumed constant mortality | | |
| Deaths* | Death in hospital *2.2 (adjusted for community deaths), *0.9 (adjusted for influenza activities). | | | | |
| DALYs averted | Used GBD 2010 DALY weights for severe and non-severe ALRI. DALYs were undiscounted and non-age weighted. | | | | |
| Abbreviations: ALRI, acute lower respiratory infection; DALY, disability-adjusted life year; GBD, Global Burden of Disease; RSV, respiratory syncytial virus; UA, University of Antwerp in partnership with the London School of Hygiene & Tropical Medicine.  *Note: All major disease burden inputs based on the Shi et al. systematic review* [7]*.* *Please refer to Table 3 for the parameter values used in each of the models.*  ^§^ Since the gender-specific data were provided at 5-year intervals, two linear curves were fitted to interpolate the monthly mortality rate using the wpp2017 package in R.  **Adjustment factors were derived from Shi et al., 2017* [7]*, which suggests scaling up hospital deaths by a factor of 2.2 to account for community deaths and scaling down the all deaths by a factor of 0.9 to account for misdiagnosis of deaths due to influenza.* | | | | | |
| **Appendix Table 3.1. List of 73 Gavi countries included in the analysis (by World Bank 2017 income group classification).** | | | | | |
| **Low-income countries**  **(N = 31)** | | | **Lower-middle-income countries**  **(N = 37)** | | **Upper-middle-income countries**  **(N = 5)** |
| Afghanistan | | | Armenia * | | Angola |
| Benin | | | Bangladesh | | Azerbaijan* |
| Burkina Faso | | | Bhutan | | Cuba* |
| Burundi | | | Bolivia | | Georgia* |
| Central African Republic | | | Cambodia | | Guyana |
| Chad | | | Cameroon | |  |
| Comoros | | | Congo, Rep. | |  |
| Korea, Dem. People's Rep. | | | Côte d'Ivoire | |  |
| Congo, Dem. Rep. | | | Djibouti | |  |
| Eritrea | | | Ghana | |  |
| Ethiopia | | | Honduras | |  |
| Gambia, The | | | India | |  |
| Guinea | | | Indonesia | |  |
| Guinea-Bissau | | | Kenya | |  |
| Haiti | | | Kiribati | |  |
| Liberia | | | Kyrgyz Republic* | |  |
| Madagascar | | | Lao PDR | |  |
| Malawi | | | Lesotho | |  |
| Mali | | | Mauritania | |  |
| Mozambique | | | Mongolia | |  |
| Nepal | | | Myanmar | |  |
| Niger | | | Nicaragua | |  |
| Rwanda | | | Nigeria | |  |
| Senegal | | | Pakistan | |  |
| Sierra Leone | | | Papua New Guinea | |  |
| Somalia | | | Moldova* | |  |
| South Sudan* | | | São Tomé and Principe | |  |
| Togo | | | Solomon Islands | |  |
| Uganda | | | Sri Lanka | |  |
| Tanzania | | | Sudan | |  |
| Zimbabwe | | | Tajikistan* | |  |
|  | | | Timor-Leste | |  |
|  | | | Ukraine* | |  |
|  | | | Uzbekistan* | |  |
|  | | | Vietnam | |  |
|  | | | Yemen, Rep. | |  |
|  | | | Zambia | |  |

**Countries with missing country-specific RSV incidence estimates Shi et al., 2017 [7].*

**Appendix Table 3.2. Number of countries modeled to introduce maternal RSV vaccine by year with corresponding birth cohort.**

| **Vaccine introduction year** | **2023/2024** | **2025/2026** | **2027/2028** | **2029 and later** |
| --- | --- | --- | --- | --- |
| Number of countries expected to introduce during this time | 16 | 39 | 14 | 4 |
| Expected cohort of eligible pregnant women in year 2023 | 15,015,380 | 18,457,389 | 47,651,364 | 2,611,303 |

**Appendix Table 3.3. Countries with missing disease burden (incidence of RSV-ALRI per 1,000 children under age 5) data and supplementary values used for modeling.**

| **Country** | **PATH** | **UA** |
| --- | --- | --- |
| Armenia | 40.8 | 56.6 (Iran) |
| Azerbaijan | 85.5 | 54.8 (Iraq) |
| Cuba | 85.8 | 40.3 (Costa Rica) |
| Georgia | 40.8 | 56.6 (Iran) |
| Kyrgyz Republic | 40.8 | 61.3 (Jordan) |
| Moldova | 40.8 | 52.8 (Average of Gavi-supported non-missing countries) |
| South Sudan | 94.0 | 56.9 (Sudan) |
| Tajikistan | 40.8 | 57.2 (Yemen) |
| Ukraine | 40.8 | 52.8 (Average of Gavi-supported non-missing countries) |
| Uzbekistan | 40.8 | 61.3 (Jordan) |

**Appendix Table 4**

**Appendix Table 4.1. Aggregate estimates of disease burden and health impact in 73 Gavi-supported countries for year 2023–2035, by scenario. (PLEASE REFER TO EXCEL FILE)**

**Appendix Table 4.2. Estimates of disease burden and health impact across 73 Gavi-supported countries by year. (PLEASE REFER TO EXCEL FILE)**

**Appendix Table 4.3. Estimates of disease burden and health impact by country in year 2035 by country. (PLEASE REFER TO EXCEL FILE)**

**Appendix Table 4.4. Estimates of vaccine coverage and target population for select years. (PLEASE REFER TO EXCEL FILE)**

**Appendix Figure 1. Schema of basic model structure.**

Abbreviations: ALRI, acute lower respiratory infection; RSV, respiratory syncytial virus.

The basic structure is common to both models. All models follow a cohort of pregnant women who are eligible to receive the vaccine. The intervention and no-intervention groups give birth to babies who may or may not be protected against RSV during early infancy. Infants may or may not get RSV.

* PATH’s model includes severe cases that may lead to hospital admissions and no hospital admission; however, UA’s model did not include severe cases that were not hospitalized due to insufficient age-specific data (see Table 2).

^ Community death was calculated using an adjustment factor (see Appendix Table 2).

**Appendix Figure 2. Vaccination coverage estimation and assumptions.**

Given that RSV MI is expected to happen during ANC visits, it is critical to understand contacts with the health system and their timing.  Essentially, we utilize Demographic and Health Surveys (DHS) data to understand the number of women that attend one, two, three, and four ANC visits. The DHS also provides timing of the first ANC visit. We then used FANC guidance (WHO) to model the timing of latter visits in the absence of any data on timing. Once we determine the number of women that attend an ANC visit in the appropriate period for RSV maternal vaccination, we apply a discount factor based on the provision of other ANC interventions. Steps used to derive vaccination coverage are listed in the figure below.

| **ANC visit** | **Eligible for vaccination** | **Visited ANC** | **Vaccinated** | **Ineligible for receiving vaccine in the next visit** |
| --- | --- | --- | --- | --- |
| **ANC1** | All pregnant women | Share of pregnant women who visited ANC1 during vaccination window * **A** * ANC1 coverage rate | **B** * Probability of receiving vaccine | Women who are no longer pregnant at next visit (for example, women who visit ANC1 late in pregnancy) + **C** |
|  | **= [A]** | **= [B]** | **= [C]** | **= [D]** |
| **ANC2** | Number of pregnant women still eligible at ANC2 visit, i.e., [A–D] | **E** * ANC2 coverage rate | **F** * Probability of receiving vaccine | Women who are no longer pregnant at next visit (for example, women who visit ANC2 late in pregnancy) + **G** |
|  | **= [E]** | **= [F]** | **= [G]** | **= [H]** |
| **ANC3** | Number of pregnant women still eligible at ANC3 visit, i.e., [E–H] | **I** * ANC3 coverage rate | **J** * Probability of receiving vaccine | Women who are no longer pregnant at next visit (for example, women who visit ANC3 late in pregnancy) + **K** |
|  | **= [I]** | **= [J]** | **= [K]** | **= [L]** |
| **ANC4** | Number of pregnant women still eligible at ANC3 visit, i.e., [I–L] | **M** * ANC4 coverage rate | **N** * Probability of receiving vaccine | Women who are no longer pregnant at next visit (for example, women who visit ANC3 late in pregnancy) + **O** |
|  | **= [M]** | **= [N]** | **= [O]** | **= [P]** |

Abbreviation: ANC, antenatal care.

$$\boldsymbol{Overall vaccine coverage rate}=\frac{[C]+[G]+[K]+[O]}{[A]}$$

***Assumptions and definitions used:***

**[A]:** Number of pregnant women = Live births ^[1]^ + Still births ^[2]^.

^[1]^ Country-specific annual population estimates from the UN World Population Prospect (2017).

^[2]^ Country-specific estimates of still birth rates from Lawn et al., 2016.

Other assumptions: No other deaths related to pregnancy and childbirth as well as abortions and fetal loss.

**[B]:** Share of pregnant women visiting ANC1 by month was derived from DHS as the total share of ANC1 visitors. Pregnant women visiting for ANC1 in a given month were evenly divided by four to estimate proportion by week (assuming 4 weeks in a month).

**[C]:** Probability of receiving vaccine: All women attending ANC are not expected to receive the vaccine, thus we discount the coverage to reflect factors such as vaccine availability and acceptance. The probability of receiving the vaccine was derived using DHS data. Most DHS report on number of services the ANC visitors received during their last pregnancy. We consider the number of services received by ANC visitors to proxy availability, accessibility, and acceptability of health services. We thus used the weighted average of the share of ANC visitors who received the respective services during ANC visits to adjust the ANC coverage rates. The basic sets of ANC services included in the DHS are (1) informed of pregnancy complications, (2) weight measured, (3) height measured, (4) blood pressure taken, (5) urine sample taken, and (6) blood sample taken.

**[E]/ [I]/ [M]:** Number of women expected to visit ANC 2, 3, or 4 based on FANC timing guideline. We also assume that those who visited ANC1 at any time during the pregnancy would come back for the next visit (ANC2) after xx weeks. All women attending ANC2 will comeback for ANC3 in xx weeks, and similarly, women attending ANC3 will visit ANC4 in xx weeks. Half of the follow-up visitors were assumed to visit at the exact week, 25% of follow-up visitors would visit before 1 week, and the remaining 25% of follow-up visitors would visit 1 week after the scheduled visit.

**[F]/ [J]/ [N]:** Estimates of ANC1 coverage and ANC4 coverage were derived from DHS for the most recent year. Countries with missing DHS information were supplemented with additional sources including the United Nations Children’s Fund, Multiple Indicator Cluster Survey, etc. Coverage rates for ANC2 and ANC3 were inferred simply by using a stepdown approach from ANC1 and ANC4 estimates.

**Appendix Figure 3. Distribution of health impact of RSV MI in year 2035, under baseline scenario.**


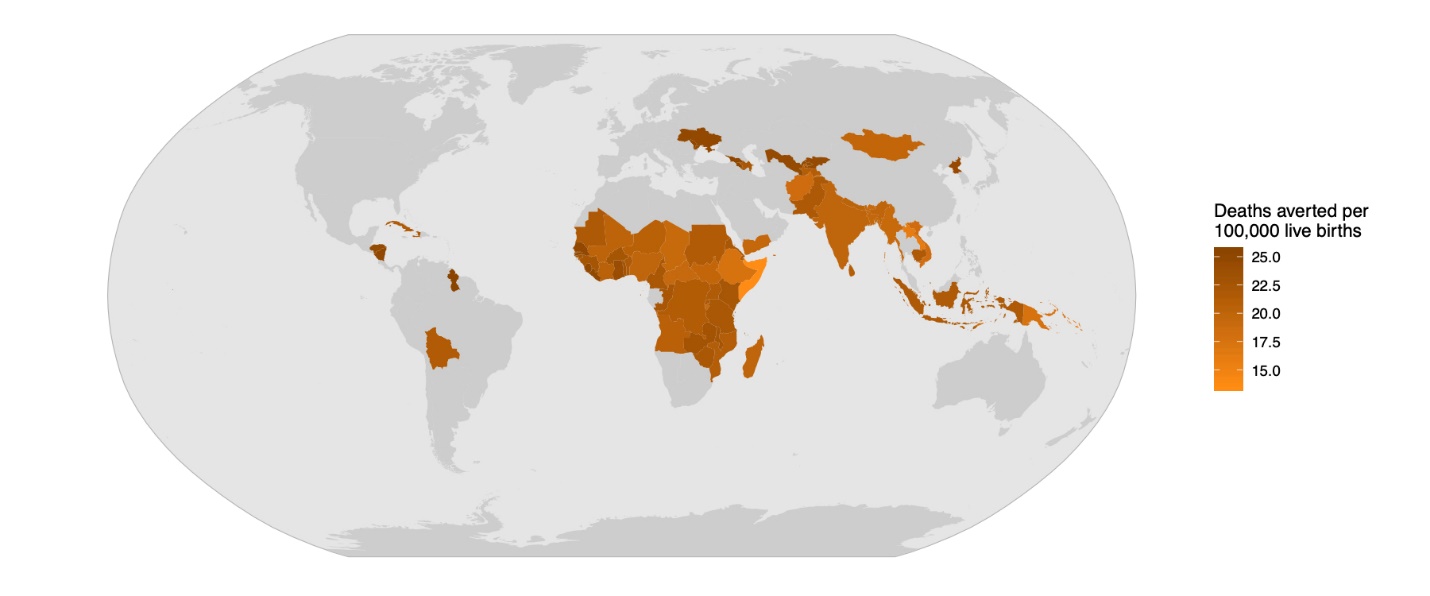
*Panel a: Distribution of deaths averted per 100,000 live births in year 2035 under baseline scenario (average estimates across two models).*


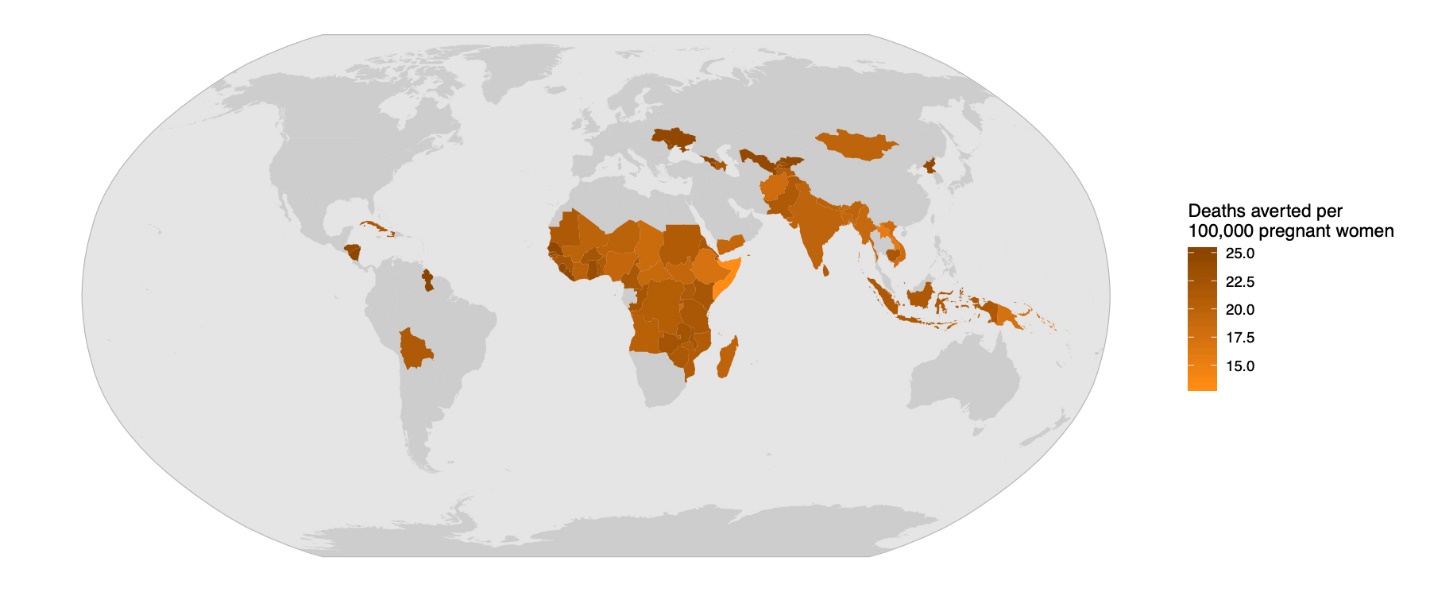


*Panel b: Distribution of deaths averted per 100,000 pregnant women in year 2035 under baseline scenario (average estimates across two models).*
